# A perforated sigmoid colon cancer initially diagnosed as a tubo‐ovarian abscess: A teaching case

**DOI:** 10.1002/ccr3.5982

**Published:** 2022-06-26

**Authors:** Asieh Maleki, Parvaneh Layegh, Seyedeh Hoda Seddighian, Maedeh Khosravi, Mona Ariamanesh, Mansoureh Dehghani

**Affiliations:** ^1^ Omolbanin Hospital Mashhad University of Medical Sciences Mashhad Iran; ^2^ Department of Radiology, School of Medicine Mashhad University of Medical Sciences Mashhad Iran; ^3^ Women's Reproductive Health Research Center Mashhad University of Medical Sciences Mashhad Iran; ^4^ Department of Pathology Neyshabur University of Medical Sciences Neyshabur Iran; ^5^ Cancer Research Centre Mashhad University of Medical Sciences Mashhad Iran

**Keywords:** adnexal mass, colon cancer, tubo‐ovarian abscess

## Abstract

Given the wide range of differential diagnoses for adnexal masses, the key issue is the correct assessment of the initial location to rule out malignant or emergency cases. Here, we report a case of perforated sigmoid colon cancer initially diagnosed as a tubo‐ovarian abscess.

## Graphical abstract

Colon cancer is a malignancy that can be confused with various conditions such as abscesses and inflammations, due to the possibility of involvement in different areas of the abdomino‐pelvic cavity. It is important to consider when facing abdomino‐pelvic findings, as early diagnosis will lead to a less morbid, curative treatment.

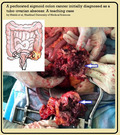

## 1 INTRODUCTION

Adnexal masses (masses that originate from the ovaries, fallopian tubes, and adjacent structures) are seen in women of all ages. The etiology and prevalence vary with age. A wide range of these masses are benign and asymptomatic and are found randomly on examination or through imaging. Accordingly, the true prevalence of these masses in the general population is unknown.^1^, ^2^ The origin of these masses can be categorized into two types: gynecological and non‐gynecological. Gynecological causes include functional and physiological cysts, endometriosis, leiomyoma, tubo‐ovarian abscesses, hydrosalpinx, and para tubal cysts, benign and malignant ovarian neoplasms. Non‐gynecological causes entail gastrointestinal masses and cancers, abscesses and tumors of the appendix and bladder and ureteral diverticulum, peritoneal and omental cysts and various retroperitoneal lesions.^3^, ^4^ Tubo‐ovarian abscess, one of the gynecological causes of adnexal masses, is almost always a complication of pelvic inflammatory disease that can be diagnosed with symptoms including the presence of a mass in the adnexa, fever, increased white blood cells, abdominal and pelvic pain, or vaginal discharge. Nonetheless, the symptoms of the disease are very diverse, which makes the diagnosis difficult. Accordingly, it can be highly crucial to obtain an accurate history and perform thorough physical examinations, including abdominal exams, speculum examination, and bimanual examination.^5^, ^6^, ^7^

Given the wide range of differential diagnoses and the lack of need to treat many benign cases, the key issue is the correct assessment and accurate diagnosis of the initial location of the mass to rule out malignant or emergency cases. This constitutes the primary challenge when dealing with these masses. Masses of gastrointestinal origin are among the most common non‐gynecological causes that should always be considered as a differential diagnosis.^4^, ^8^

Here, we report a case of perforated sigmoid colon cancer in a 45‐year‐old female patient which was preoperatively diagnosed as a tubo‐ovarian abscess.

## 2 CASE REPORT

A 45‐year‐old female, P3L3/ND (3 previous normal pregnancies/3 children), was referred to the gynecology department with abdominal pain in the left lower quadrant (LLQ) region, fever, and chills.

The patient had also referred to the emergency room a week earlier with a similar abdominal pain, without fever. At the time, an ultrasound imaging report had described a 4‐centimeter heterogeneous area in left ovary, and she was discharged with a prescription of cefixime based on the diagnosis of hydrosalpinx on ultrasound and the absence of fever.

The pain intensified during the 2 days, and she also mentioned the reduction of appetite and chronic constipation over the last few months. There was no nausea and vomiting. She had no medical illness history, no surgical history, and no remarkable drug or family history.

The patient was ill and febrile during the general examination. Blood pressure was 100/60 mmHg, heart rate was 110 beats/minute, and body temperature: 39°C. On physical examination, the patient's abdomen was soft and had no tenderness or rebound positivity, and the lungs were clear. On genital examination with a speculum, the appearance of the cervix was normal, mucopurulent secretions were seen in the cervix, and cervical motion tenderness was positive. The patient underwent an ultrasound investigation which demonstrated a normal uterus with endometrial thickness of 10 mm and a round hypoechoic cystic lesion containing hyperechoic areas with approximate dimensions of 61 × 82 mm on the left side of the pelvis. Significant inflammation and edema in the surrounding soft tissue with possibility of tubo‐ovarian abscess on the left side were reported, while the right ovary was normal.

According to the examinations and the results of ultrasound imaging, there was a suspicion of pelvic abscess, and the patient was hospitalized with a diagnosis of PID and tubo‐ovarian abscess. She underwent antibiotic therapy with ampicillin 2 g QID, clindamycin 900 mg TDS, and gentamicin 80 mg TDS.

**FIGURE 1:**
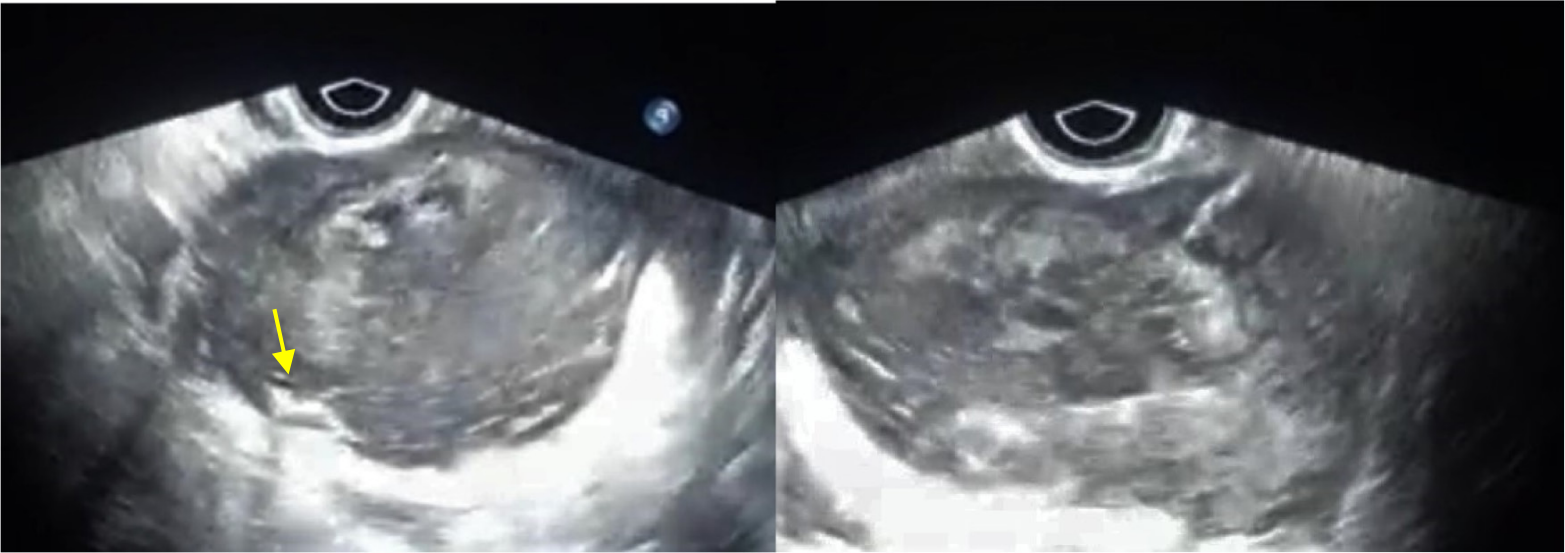
A complex mass with internal echo in the cystic component and air shadow (arrow) in the left side of the pelvic, which encased the left ovary

As the patient's fever did not alleviate after 48 h of antibiotic administration, the antibiotic regimen was changed to Imipenem and the patient was a candidate for repeated ultrasound evaluation and Ultrasound Guided Abscess Drainage. The next ultrasound assessment revealed a heterogeneous cystic structure with internal echoes and gas foci in the left adnexa extending to the posterior cul‐de‐sac with dimensions of 100 × 41 mm, along with evidence of fat stranding and increased thickness of the adjacent intestine (Figure 1). The attempt to drain the abscess, however, proved unsuccessful.

**FIGURE 2:**
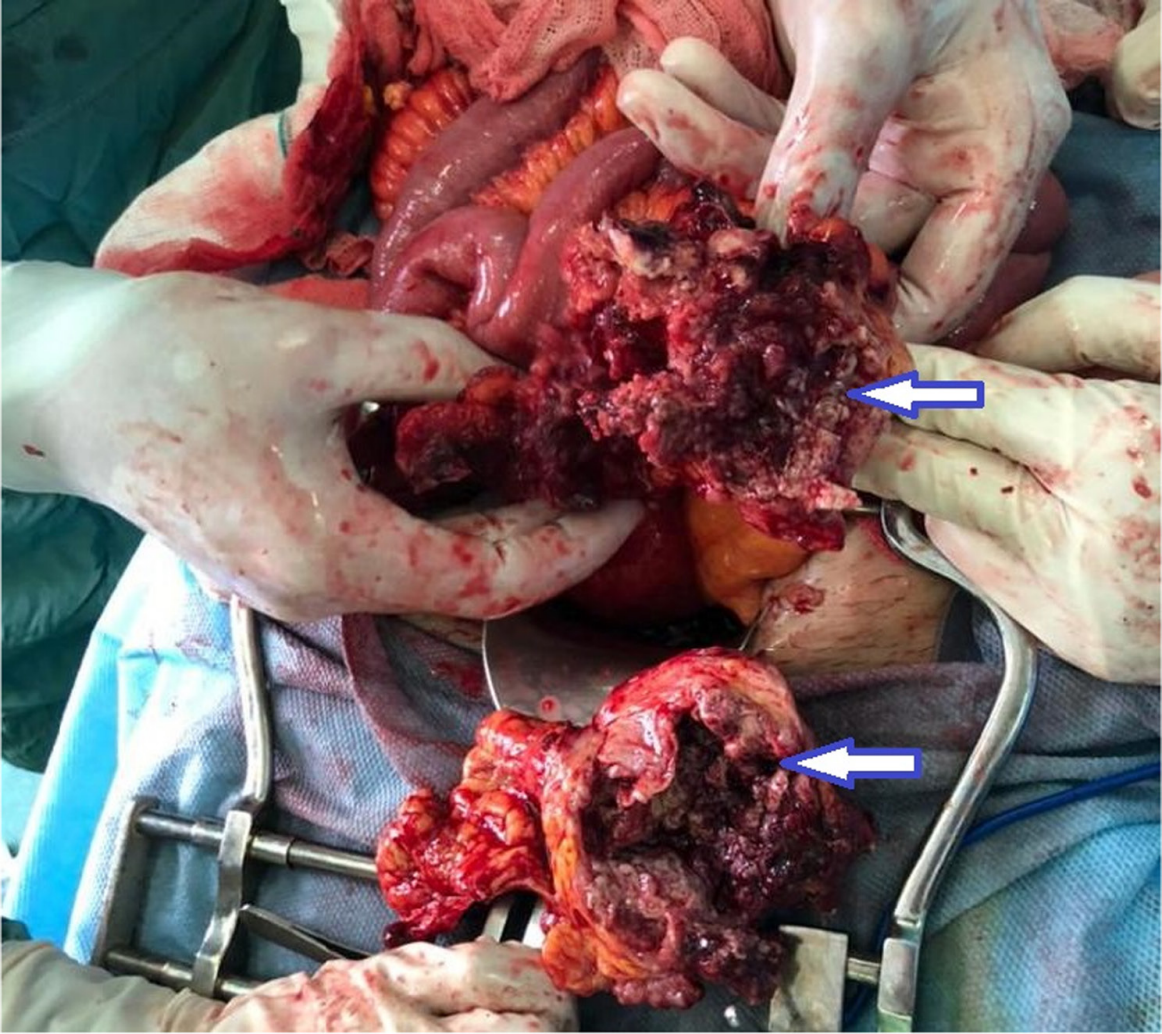
Intraoperative finding included a necrotic perforated sigmoid mass (arrows)

**FIGURE 3:**
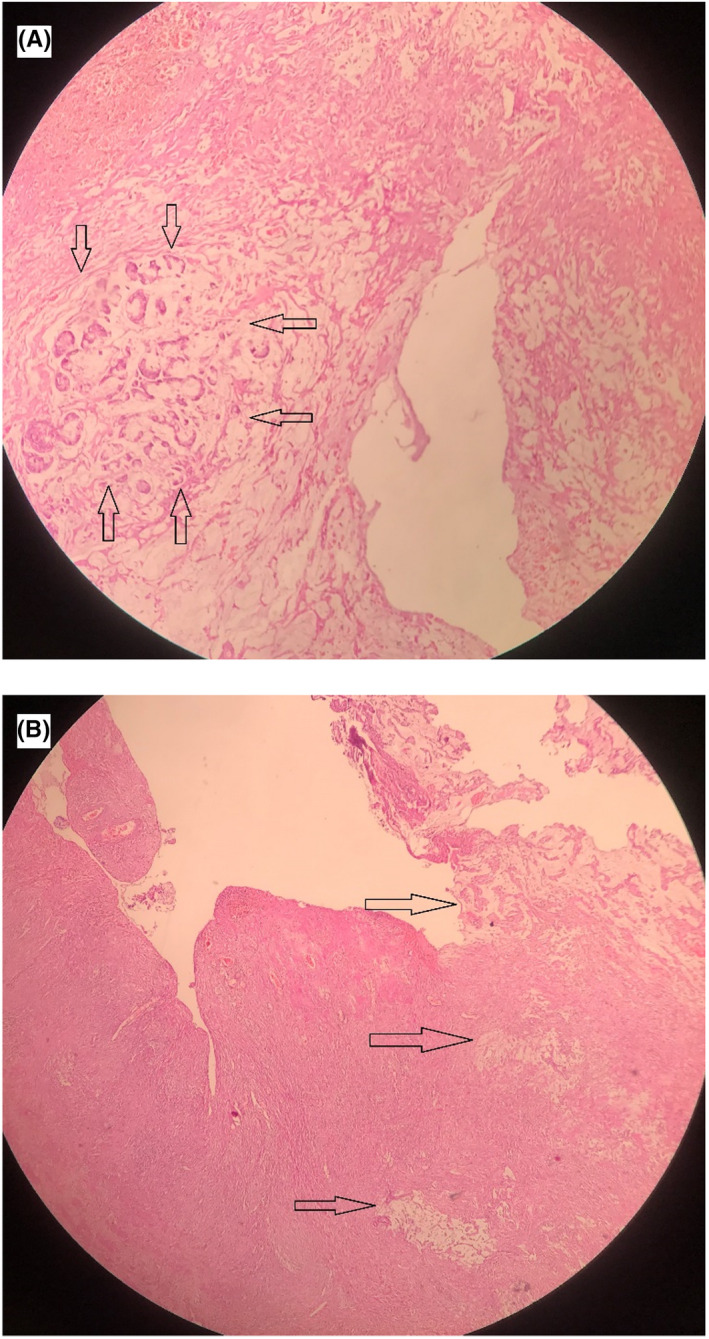
Microscopic evaluation of the permanent specimen demonstrating carcinomatous involvement in fallopian tube muscular layer (A) and carcinomatous invasion to the surface and parenchyma of the ovary (B)

Due to the lack of response to the treatments, and with an initial impression of tubo‐ovarian abscess, the patient underwent midline laparotomy in the gynecology operation room. When the abdominal wall was opened with a median incision, an extensive adhesion of the intestines and omentum to the posterior wall of the uterus and the left adnexa was observed. After removing the adhesions, a 10 × 15 cm necrotized, walled‐off area of colon on the left side and behind of the uterus was present (Figure 2). Frozen section of the tumoral region was obtained and sent for pathologic review, which resulted in the diagnosis of sigmoid colon adenocarcinoma. Oncosurgery consult was requested intra‐operatively and due to sigmoid perforation and the frozen section results, showing malignant mucinous adenocarcinoma, sigmoidectomy and colostomy were performed using the Hartman method. Microscopic evaluation of permanent specimens also confirmed the diagnosis and reported the TNM staging pT4N3^9^ (Figure 3). Considering the malignant diagnosis, abdominal and pelvic computed tomography (CT) scans were performed in order to detect possible metastasis, which indicated negative for distant metastasis (stage III). The postoperative period was uneventful, and the patient was discharged in good general condition with colostomy, being referred to outpatient oncology clinic for further management.

## 3 DISCUSSION

Colorectal cancer (CRC) is a common and deadly disease and one of the three leading causes of death in women, according to the statistics reported by the World Health Organization.^10^ Most cases of CRC are asymptomatic or with minimal symptoms for a considerable time at the beginning of the disease. Patients may present with abdominal pain, change in bowel habits, hematochezia/melena, iron deficiency anemia, general weakness, and weight loss. Signs of tumor‐related obstruction may also be present. Apart from obstructive symptoms, other presentations are not associated with stage of disease or prognosis.^11^ Considering the fact that many of the primary symptoms are nonspecific, the present patient might have had these symptoms for some time without realizing its relevance. There was also a history of reduction of appetite and chronic constipation of which the patient was ignorant until the second referral to the hospital.

Site of the tumor is a major contributor to the clinical presentation. Cancers of the right colon are often exophytic and commonly associated with anemia due to occult blood loss. Cancers of the left colon and sigmoid colon are often deeply invasive and annular. Obstruction, perforation, peritonitis, rectal bleeding, and alteration in bowel habits are common in these cases.^11^, ^12^, ^13^ These symptoms with an adnexal mass finding on imaging can result in a confusion for a tubo‐ovarian abscess.

Most colorectal cancer is associated with symptoms such as nausea, vomiting, and constipation, before resulting in perforation and peritonitis and it is uncommon for the colon to be perforated before the complete intestinal obstruction. As such, these symptoms, along with imaging results of adnexal masses, can lead to the suspicion and misdiagnosis of tubo‐ovarian abscess, which was reported in a similar case study by Sayan et al.^14^ In that case, similar to our patient, a complex adnexal mass was presumed to be a tubo‐ovarian abscess preoperatively, which was diagnosed later as a large perforation area on sigmoid colon with omental reaction.

The evaluation of colorectal cancers should include pan‐body computed tomography (CT) scan. Nevertheless, considering the fact that the evaluation of acute abdominal pain, especially in the Department of Gynecology and in patients with suspected gynecologic problems, begins with ultrasound^15^, ^16^; if there is a finding that leads to the diagnosis of an acute event, the possibility of performing surgery before wasting time with CT scan, especially in centers with limited access, is a possible process that can lead to surgery with incorrect initial diagnosis. To prevent such cases, this approach requires more precision and consideration of the degree of reliability and other differential diagnoses.

Reports of misdiagnosis cases of gastrointestinal pathologies with adnexal masses have been published, indicating the great importance of using diagnostic tests and imaging along with principled physical examination. However, misdiagnosis of appendix pathologies is more relevant to adnexal diseases. In the cases reported by Cristian et al., Hajiran et al., and Kumar et al., the patients were presented with the symptoms of adnexal mass. However, after final diagnostic examinations, the presence of mucinous adenocarcinoma of the appendix in these patients was confirmed.^17^, ^18^, ^19^

Whereas transabdominal ultrasound is not routinely used in the diagnosis and staging of colon cancer, recent technology advances as well as the highly cost‐effective and noninvasive nature of this technique has prompted new interest, with much less excuse to call it a weak assessment tool. Although still investigational, transabdominal ultrasound could prove to be an important alternative to existing standard protocols.^12^, ^20^

Mucinous adenocarcinoma accounts for 10%–20% of colorectal adenocarcinomas, which are common in women and young patients. Studies in patients with this type of colorectal cancer have demonstrated that despite the relatively poorer prognosis, systemic chemotherapy and surgery have had positive effects on the treatment process of patients. To achieve an appropriate therapeutic response, it is important that patients are identified and treated in a timely manner.^21^, ^22^ As Emergency surgery for colon cancer is associated with higher risk of recurrence and death, colon cancer awareness in the face of various manifestations of the disease in order to reduce emergency cases is warranted.^23^

## 4 CONCLUSION

Given the variety of symptoms in patients with pelvic masses and the risks of misdiagnosis, along with the importance of a timely diagnosis of malignant or emergent situations, it is recommended that all diagnostic possibilities be considered to rule out irrelevant diseases before the patients with pelvic masses are treated.

## AUTHOR CONTRIBUTIONS

Authors AM, PL, and SHS contributed to conception, design, and drafting of the manuscript. Authors MK, MA, and MD contributed to data collection. MA contributed to providing microscopic data and imaging.

## CONFLICT OF INTEREST

The authors have no conflict of interest to declare.

## ETHICAL APPROVAL

Written informed consent was obtained from the patient to report the case, and the manuscript was approved at the Ethics Committee of Mashhad University of Medical Sciences.

## CONSENT

Published with written consent of the patient.

## Data Availability

All data generated during this study can be accessed through direct communication with the corresponding author and the agreement of all research team members.
